# Bell Clapper Testis, Torsion, and Detorsion: A Case Report

**DOI:** 10.1155/2011/631970

**Published:** 2011-09-19

**Authors:** F. Khan, Okwudili Muoka, G. M. Watson

**Affiliations:** Department of Urology, NHS Eastbourne District General Hospital, Eastbourne BN21 2UD, UK

## Abstract

We are presenting a common but interesting case of testicular torsion. Torsion is a common important urological emergency. History and examination is important for diagnosis. Urgent testicular exploration is an important message if in doubt.

## 1. Introduction

Testicular torsion is the most common cause of acute scrotal pain in prepubertal and adolescent boys. Intermittent testicular torsion should be considered in all young males with a history of scrotal pain and swelling. Intrauterine torsion of the testis, although rare, is being recognised with increasing frequency [1]. 

 Late presentation to hospital is the major cause of delay in diagnosis and mostly leads to orchidectomy in such patients [2]. Intermittent testicular torsion (ITT) is a poorly characterized condition but harbours potentially serious implications with regard to testicular viability.

## 2. Case History

A 24-year-old male was referred for left groin pain. It was severe and sudden in onset for six to seven hours with no urinary symptoms or urethral discharge. General examination including testicles was normal. He discharged from A&E after reassurance.

 He visited emergency department again in 10 days time with left testicular pain. There were no urinary symptoms or history of trauma. Urine and bloods were normal. Diagnosis of epididymo-orchitis was made on clinical as well as radiological findings and he was discharged from hospital. On follow up outpatient visit, left testis was small, high riding, and indurated. An urgent ultrasound confirmed dead testis. He was booked for a left orchidectomy and right orchidopexy.

Few weeks later, he was admitted with right testicular pain and on exploration Bell Clapper testis was found. Right testis was viable and left one was dead. A 3-point fixation of right viable testis and left orchidectomy of dead testis was performed.

## 3. Discussion

Testicular torsion is the most common paediatric genitourinary emergency [3] and probably the second most common surgical emergency in the adolescent age group. It occurs in approximately one in 4000 males under 25 years of age [4]. If prolonged, torsion results in infarction, but even those testes which are salvaged by surgery may undergo atrophy [5]. Human testes occasionally survive up to 10 hours of torsion; however, viability is considerably reduced after 4–6 hours of ischemia [6].

Diagnosis of torsion is mainly clinical. Doppler ultrasound scan can be helpful in suspected cases. The sensitivity of colour doppler ultrasound scan ranges from 89% to 100% [7]. Theoretically the sensitivity of doppler scanning may be lower in incomplete or intermittent testicular torsion, in both of which flow can be normal. 

 Early diagnosis and definitive management are the keys to avoid testicular loss. All prepubertal and young adult males with acute scrotal pain should be considered to have testicular torsion until proven otherwise. The finding of an ipsilateral absent cremasteric reflex is helpful, but not diagnostic.

 Treatment involves rapid restoration of blood flow to the affected testis. Manual detorsion by external rotation of the testis can be successful, but restoration of blood flow must be confirmed following the manoeuvre. Surgical exploration provides definitive diagnosis and management according to findings.

The Bell Clapper deformity is one of the causes for the testicular torsion. In this condition, testis lacks a normal attachment to tunica vaginalis and hangs freely. If history and examination suggest torsion, urgent testicular exploration is the only best way to proceed. The learning point in our case report is every case of testicular pain in children or adolescent should be treated as testicular torsion until proved otherwise. Examination should include testicles, if the patient is referred with groin pain. Surgical exploration should be the first line of management in suspected cases.
